# The Hyderabad Commission on the Action of Chloroform

**Published:** 1889-11-30

**Authors:** 


### THE HYDERABAD COMMISSION ON THE ACTION OF CHLOROFORM.

It will be in recollection that the Government of H.H. the Nizam appointed a commission to investigate the action of chloroform. The members of the commission were Dr. Hebrin and Messrs. Charmarette and J. Kelly, L.M.S., acting under the residency surgeon of Hyderabad, Dr. E. Laurie. One hundred and forty-one dogs were experimented upon, and the experiments were divided into four series : 1. Anæsthetised until death took place. 2. Large doses given. 3. Gradually anæsthetised with drachm doses. 4. Rapidly with concentrated chloroform, without admixture of air. The report is most elaborate, and the conclusions the commission arrived at are that the main point in chloroforming is to watch the breathing; that artificial respiration is useless, if breathing has ceased, more than twenty seconds ; that it is impossible for chloroform to kill so long as respiration is kept up ; that cardiac syncope does not occur. The residency surgeon, Dr. Laurie, in reviewing the report of the commission, observes : "It was not found that chloroform produced any depressant action on the heart, and that the heart never became affected until after the breathing had stopped." When once anæsthesia was produced, provided the respiration was kept up, there was no cumulative effect, and the chloroform was rapidly eliminated. This is the original observation, which embodies the pith of the commission's report. This tallies exactly with the experience of Dr. Laurie, who has superintended some 40,000 or 50,000 administrations of chloroform. "In London, deaths from chloroform constantly occur, but provided the administrator can swear he examined the heart and felt the pulse, they are always supposed to be accidental." And Dr. Laurie has no doubt that deaths will go on occurring until the London schools entirely change their principles and ignore the heart in chloroform administration. The rules which prevail for the administration of chloroform in the Hyderabad Medical School are as follows. Before all operations in adults a hypodermic injection of morphia is given. In this connection it is worthy of note that some American practitioners recommend a little spirit to be given before chloroform is used. This is done at Hyderabad if the patient is depressed or afraid, or the operation likely to be prolonged. The recumbent position is regarded as essential. An apparatus ought not to be used, as it takes up part of the attention required elsewhere. The admixture of air with the vapour of chloroform is ensured by an open cone with a little absorbent cotton wool inside at the apex. The more rapidly chloroform is given the better, and the quantity is not measured. In children, crying ensures free admission of chloroform into the lungs, and adults should be made to blow out hard after each inspiration. The patient is ready for the operation when winking is not produced by touching the surface of the eye. The administrator should be guided as to effect entirely by the respiration. His only object while producing anæsthesia is to see that the respiration is not interfered with. If possible, the chest and abdomen should be exposed, so that the respiratory movements may be seen. If anything interferes with the respiration, even if this occurs at the very commencement of the administration—if the breath is held, or there is stertor, the inhalation should be stopped. If the breathing becomes embarrassed, the lower jaw should be pulled or pushed from behind the angles, so that the lower teeth protrude, which raises the epiglottis and frees the larynx. If the respiration stops, artificial respiration should be commenced at once. In the hospital attached to the Hyderabad Medical School chloroform is invariably given with absolute safety by students, and "they are never allowed to examine the heart beforehand, or to feel the pulse during respiration." Now the conclusion that the heart is not primarily affected during chloroform inhalation has been much questioned in London. And it has been argued that experiments on dogs do not warrant conclusions as to effect on human beings. But it is stated in the report that the results of chloroform inhalations in dogs are nearly, if not uniformly, the same as those produced in man. The only points of difference in the effects of chloroform in the dog and in the man, are that in the latter embarrassment of the respiration is usually indicated, or preceded, by stertor, while in the dog there is no stertor or any other warning, except irregularity and shallowness, to denote that respiration is impeded. In the dog, the facial, and less frequently the thoracic, movements of respiration may continue for some seconds after air has entirely ceased to enter the lungs. In man this very rarely happens. These differences, slight as they are, are important, as they show that it is more easy to overlook the arrest of respiration in the dog than in the man, and "they may account for the errors of previous investigators who have asserted that the heart sometimes stops before the respiration in death from chloroform." Since this report was published fresh investigations have been made at Hyderabad, in the presence of an eminent London physician, and it is understood that the conclusions of the original commission have been confirmed. We await with great interest more on this important subject. His Highness the Nizam, his minister, Sir Asman Jah, the residency surgeon, Dr. Laurie, and the members of the commission richly deserve thanks for this contribution to science.

